# Association of oral diadochokinesis with activities of daily living in older adults: a cross-sectional observational study

**DOI:** 10.1186/s12903-026-08799-1

**Published:** 2026-06-03

**Authors:** Haruna Ushimura, Tomoko Yamaji, Mikie Hidaka, Hidetoshi Miyata, Kazuo Iwasa, Shihomi Sakurai

**Affiliations:** 1 Faculty of Nursing, Ishikawa Prefectural Nursing University, 1-1 Gakuendai, Kahoku, 929-1210 Japan; 2 Tokyo Medical University School of Nursing, Shinjuku, Japan; 3 Miyata Dental Office, Kanazawa, Japan; 4 Department of Public Health, Department of Health and Medical, Ishikawa Prefecture Dental Association, Kanazawa, Japan; 5 Department of Health and Medical Sciences, Ishikawa Prefectural Nursing University, Kahoku, Japan

**Keywords:** Older adults, Oral diadochokinesis, Masticatory function, Activities of daily living, Tongue function

## Abstract

**Background:**

Deterioration in tongue and masticatory functions is linked to frailty and disability-related functional abilities among older adults. This study aimed to examine the association between tongue and masticatory functions and activities of daily living (ADL) of older adults with varying levels of independence.

**Methods:**

This cross-sectional study included 100 participants aged 70–85 years old. ADL was assessed using the Barthel Index (BI), and 79 and 21 participants were classified into the good ADL (BI ≥ 90) and poor ADL (BI < 90) groups, respectively. Tongue and masticatory functions were evaluated using oral diadochokinesis (ODK; /pa/, /ta/, and /ka/), tongue pressure, masticatory performance (gummy jelly test), and number of remaining teeth. Logistic regression analyses (univariate and age adjusted) and receiver operating characteristic analyses were performed.

**Results:**

The ODK scores for /pa/, /ta/, and /ka/ were lower in the poor ADL group (*p* < 0.05). After adjusting for age, ODK measures remained independently associated with poor ADL. Masticatory performance was lower in the poor ADL group; however, this association did not remain after adjusting for age. No correlation was observed between tongue pressure, number of teeth, and performance in ADL. The area under the curve (AUC) values for ODK /pa/, /ta/, /ka/, and masticatory performance were 0.66–0.67, indicating only modest discrimination between good and poor ADL.

**Conclusions:**

ODK measures (/pa/, /ta/, /ka/) were independently associated with poor ADL status after age adjustment. However, their discriminatory ability (AUC 0.66–0.67) was limited, indicating they are not suitable as standalone screening tools without longitudinal validation.

**Supplementary Information:**

The online version contains supplementary material available at 10.1186/s12903-026-08799-1.

## Background

As the global population of older adults increases, the maintenance of physical function and extension of healthy life expectancy have become critical public health concerns. These challenges are compounded by the substantial discrepancy between performance-based and self-reported functioning [1]; therefore, potential declines in activities of daily living (ADL) should be identified at an early stage. Among older adults, the decline in tongue and masticatory functions is associated with frailty and disability-related functional decline [2, 3]. The key indicators of tongue and masticatory function are oral diadochokinesis (ODK), which is measured using the speed and regularity of tongue and lip movements and is commonly used to assess oral motor function rather than general neuromotor coordination; maximum tongue pressure, which reflects tongue strength; and masticatory function indicators, such as the number of teeth and masticatory performance. Reduced tongue pressure and masticatory function may limit food intake, particularly of harder or more fibrous foods, leading to inadequate nutritional intake [4–6]. Furthermore, reduced ODK, lower tongue pressure, fewer teeth, and lower masticatory performance have been associated with sarcopenia [7–10], which is a known contributor to physical impairment and poor ADL status [11, 12].

Among community-dwelling older adults who are mostly independent, reduced masticatory performance has been associated with poorer performance in the intellectual activity domain of ADL, which includes activities such as reading books and independently managing documents [13]. In contrast, in older adults who require care or assistance, reduced tongue pressure may contribute to worsening nutritional status, as mediated by a decline in ADL [14]. To the best of our knowledge, few studies have directly investigated the association between tongue and masticatory functions and ADL in a wide spectrum of older adults, encompassing independent community-dwelling individuals and those requiring support or care. Clarifying this association may provide critical insights for the early identification of older adults at high risk of ADL decline through the evaluation of tongue and masticatory functions.

This study aimed to investigate how tongue and masticatory functions are associated with ADL among older adults with varying levels of independence.

## Methods

### Study design and participants

**Fig. 1:**
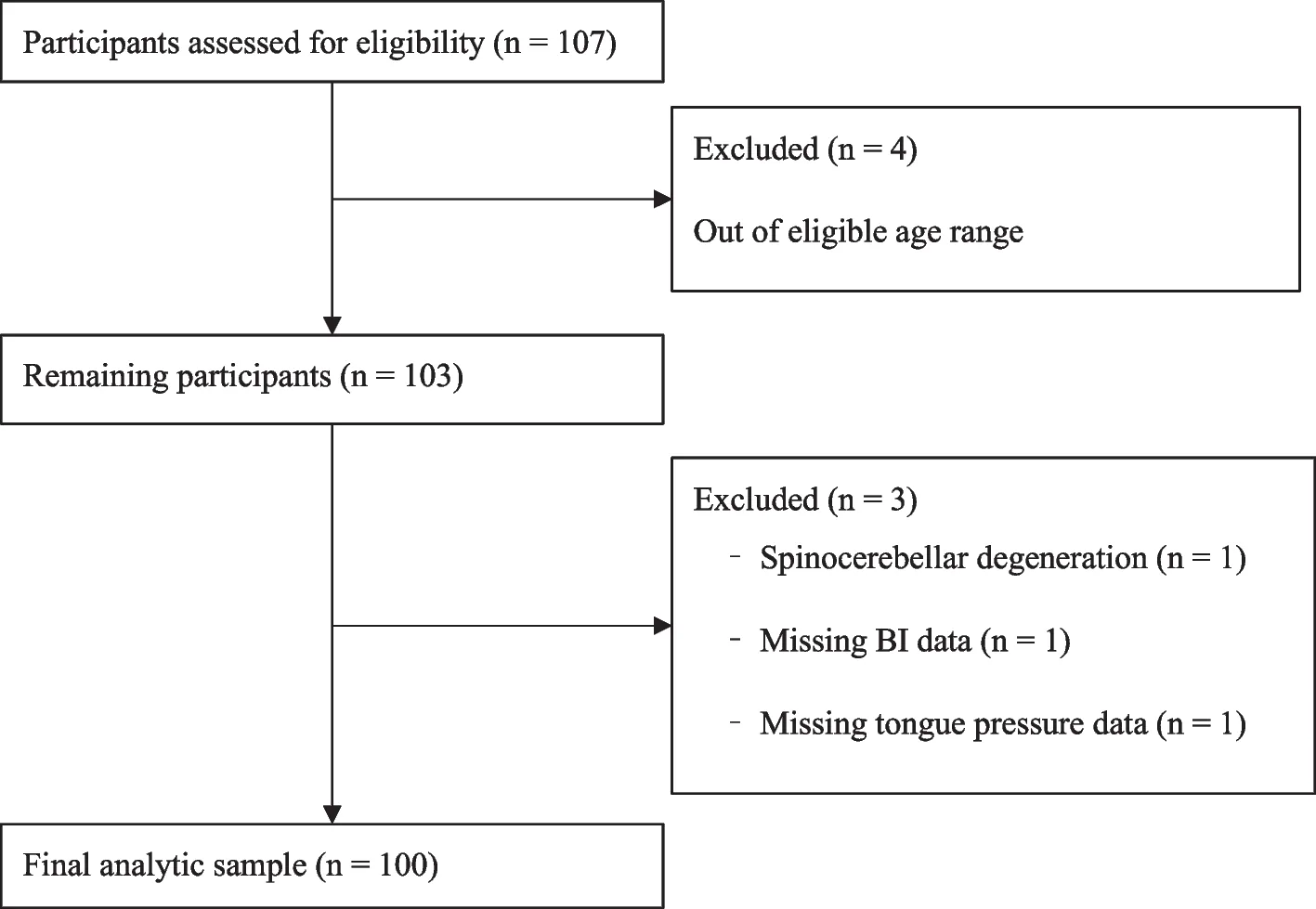
Flow diagram of participant recruitment and inclusion. A total of 107 individuals were assessed for eligibility. Four were excluded for age, and three for missing data, leaving 100 participants in the final analysis. BI, Barthel Index

This cross-sectional observational study recruited individuals aged 70–85 years. Participants were recruited from two settings: (1) community-dwelling older adults participating in community-based exercise or day-care programs and (2) older adults residing in housing-type residential care facilities that provide support services. Eligible participants were required to have the capacity to make voluntary decisions regarding study participation, withdrawal, or refusal, and the ability to understand questionnaire items and measurement procedures. The exclusion criteria were individuals undergoing treatment for oral diseases, those with a history of dysphagia or choking episodes, and those allergic to gelatin. Data were collected between October 2024 and April 2025. A total of 107 participants were initially recruited. However, four were excluded because they were outside the eligible age range (Fig. 1). Among the remaining 103 participants, three were further excluded (one with spinocerebellar degeneration, one with missing Barthel Index (BI) data, and one with missing tongue pressure data). A total of 100 individuals were included in the final analysis. No participant exhibited neurological symptoms affecting oral function, such as dysarthria or aphasia, at the time of assessment.

BI and oral function assessments were conducted by trained nurses who had received prior instructions on the assessment procedures. Each assessment was conducted independently, and assessors did not refer to the results of other measurements during data collection. All measurements for each participant were performed within a single session.

Ethical approval was obtained from the Ethical Committee of Ishikawa Prefectural Nursing University (approval no. 2024–75). All participants provided written informed consent prior to enrollment.

### ADL

ADL referred to the fundamental tasks required for independent living and was evaluated using the BI [15, 16]. The BI includes 10 items: feeding, bathing, grooming, dressing, bowel control, bladder control, toilet use, transfers (e.g., bed to chair and back), mobility on level surfaces, and climbing stairs. ADL was divided into two groups according to the BI score: good ADL (BI ≥ 90) and poor ADL (BI < 90). This cutoff was determined based on a previous report in Japanese older adults, in which median BI scores of approximately 90 marked the boundary between relatively independent and more dependent ADL status [17]. In addition, in our sample, the BI scores showed a skewed distribution with a ceiling effect, and dichotomization at a score of 90 provided a clinically interpretable distinction between those who were mostly independent and those with at least mild functional limitations.

### Tongue pressure

Tongue pressure (kPa) was measured using a tongue pressure measurement device (JMS, Osaka, Japan). For each measurement, a balloon attached to the probe was placed against the anterior palate, and the participant was instructed to press the balloon against the palate using their tongue with maximum voluntary force for 7 s. This process was repeated thrice, and the highest value was recorded.

### ODK

ODK was assessed as the number of repetitions per second of the monosyllables/pa/,/ta/, and/ka/using an automatic counter (Kenkokun Handy II; Takei Scientific Instruments Co., Niigata, Japan) to evaluate the functions of the lips, tongue tip, and posterior region of the tongue, respectively. Each syllable was measured twice, and the maximum value was used.

### Masticatory performance

Masticatory performance was assessed using gummy jellies (UHA Mikakuto Co., Osaka, Japan). The participants were instructed to chew a test gummy jelly (18.5 mm [top surface] × 21.4 mm [bottom surface] × 10.8 mm [height]) 30 times and then spit it out onto a gauze. The condition of the comminuted gummy jelly was evaluated using a 10-stage visual scale (range: 0–9), with scores 0–2 indicating decreased masticatory performance [18]. All assessments of tongue pressure, ODK, and masticatory performance were performed while the participants wore dentures.

### Number of present teeth and functional teeth

A dentist from our research team assessed the number of present and functional teeth in each participant. The present teeth were counted using photographs taken without dentures, and functional teeth were evaluated using photographs taken with dentures. Intraoral photographs with dentures were used to classify the occlusal support according to the Eichner index, which is based on the number of posterior occlusal supporting zones [19]. Category A indicates the presence of all four supporting zones; category B, partial loss of occlusal support with at least one supporting zone remaining; and category C, absence of occlusal support.

### Handgrip

Handgrip strength was measured twice for both hands in an alternating manner using a Smedley-type dynamometer (Takei Scientific Instruments Co.), and the maximum value was recorded. Handgrip weakness was defined according to the criteria of the Asian Working Group for Sarcopenia 2019 (< 28 kg for men and < 18 kg for women) [20].

### Nutritional status

Nutritional status was assessed using the Mini Nutritional Assessment®–Short form (MNA–SF) [21, 22], which consists of six items, including weight loss, mobility, psychological stress or acute disease, neuropsychological problems, and body mass index (BMI). When BMI could not be measured, calf circumference was used instead, in accordance with the standard MNA-SF procedure. Scores were categorized as normal (≥ 12), at risk of malnutrition (8-11), or malnourished (≤ 7).

### Statistical analysis

Participant characteristics were summarized using descriptive statistics. Group comparisons were conducted using the Mann–Whitney U test for continuous variables and Fisher’s exact test for categorical variables. Factors associated with poor ADL (BI < 90) were examined using binary logistic regression analysis (univariate and age-adjusted models). Considering the relatively small sample size, 95% confidence intervals (CIs) were estimated using bootstrap resampling with 1000 iterations and the percentile method. The bootstrap method, which repeatedly samples data with replacement, was used to assess variability and CIs. In the logistic regression analyses, the dependent variable was coded as 1 for BI ≥ 90 (good ADL) and 0 for BI < 90 (poor ADL). For ease of interpretation, we re-expressed the regression coefficients and reported odds ratios (ORs) as the odds of poor ADL per unit decrease in each explanatory variable: one repetition per second for ODK, 1 kPa for tongue pressure, one score for masticatory performance, and one tooth for the number of teeth. Multivariable models were adjusted only for age due to limited events (poor ADL, *n* = 21), precluding further adjustment according to the events-per-variable guidelines (~ 10 events/variable). Missing data were handled via a complete-case analysis; all the 100 participants had complete data for primary analyses. As a sensitivity analysis, we examined the association between ODK and ADL using multiple linear regression, with BI as a continuous variable and adjusting only for age.

Receiver operating characteristic (ROC) analyses were additionally conducted as exploratory analyses to examine the discriminative ability of tongue and masticatory functions in detecting poor ADL (BI < 90) and to obtain indicative cutoff values. A two-sided *p* < 0.05 was considered statistically significant. All analyses were performed using the SPSS Statistics software (version 29; IBM Corp., Armonk, NY, USA).

## Results

### Participant characteristics

The overall sample included 35 men and 65 women (median age: 80.0 years) (Table 1). Among the participants, 79 and 21 were classified into the good ADL (BI ≥ 90) and poor ADL (BI < 90) groups, respectively. Handgrip weakness was present in 31 (39.2%) and 19 (90.5%) participants in the good and poor ADL groups, respectively. The prevalence of handgrip weakness was higher in the poor ADL group than that in the good ADL group (*p* < 0.01).

**Table 1:** Characteristics of participants according to ADL status

Variables	Good ADL (BI ≥ 90, *n* = 79)	Poor ADL (BI < 90, *n* = 21)	*p*-value	
Age, years, median (IQR)	80.0 (76.0–82.0)	80.0 (76.5–83.0)	0.44	^a^
Sex, women, n (%)	55 (69.6%)	10 (47.6%)	0.07	^b^
Diabetes, n (%)	18 (22.8%)	6 (28.6%)	0.58	^b^
Hypertension, n (%)	48 (60.8%)	14 (66.7%)	0.80	^b^
Cardiovascular disease, n (%)	13 (16.5%)	4 (19.0%)	0.75	^b^
Respiratory disease, n (%)	3 (3.8%)	3 (14.3%)	0.11	^b^
Cerebrovascular disease, n (%)	10 (12.7%)	3 (14.3%)	1.00	^b^
Musculoskeletal disease, n (%)	14 (17.7%)	3 (14.3%)	1.00	^b^
Mental disorder, n (%)	2 (2.5%)	1 (4.8%)	0.51	^b^
Handgrip weakness, n (%)	31 (39.2%)	19 (90.5%)	< 0.01^*^	^b^
MNA®‐SF category				
Normal, n (%)	55 (69.6%)	11 (52.4%)	–	
At risk of malnutrition, n (%)	24 (30.4%)	10 (47.6%)	–	
Malnourished, n (%)	0 (0.0%)	0 (0.0%)	–	
Tongue pressure, kPa, median (IQR)	30.5 (24.3–38.7)	24.8 (19.2–34.7)	0.15	^a^
ODK/pa/, reps/s, median (IQR)	5.4 (4.4–6.4)	5.0 (3.5–5.8)	< 0.05^*^	^a^
ODK/ta/, reps/s, median (IQR)	5.4 (4.2–6.2)	4.0 (3.3–5.7)	< 0.05^*^	^a^
ODK/ka/, reps/s, median (IQR)	4.8 (3.6–5.8)	4.2 (2.4–5.2)	< 0.05^*^	^a^
Number of present teeth, median (IQR)	18.0 (8.0–23.0)	11.0 (2.5–22.0)	0.25	^a^
Number of functional teeth, median (IQR)	27.0 (25.0–28.0)	27.0 (22.0–28.0)	0.90	^a^
Masticatory performance, score, median (IQR)	4.0 (2.0–5.0)	2.0 (0.0–4.0)	< 0.05^*^	^a^
Wearing dentures, n (%)	55 (69.6%)	11 (52.4%)	0.19	^b^
Eichner classification				
A, n (%)	28 (35.4%)	3 (14.3%)	–	
B, n (%)	31 (39.2%)	10 (47.6%)	–	
C, n (%)	20 (25.3%)	8 (38.1%)	–	

Values are presented as median (interquartile range) or n (%)

*ADL* Activities of daily living, *BI* Barthel Index, *ODK* Oral diadochokinesis, *IQR* Interquartile range, *MNA®‐SF* Mini Nutritional Assessment–Short Form

^*^Variables with statistically significant differences between groups

^a^Mann–Whitney U test

^b^Fisher's exact test

### Association between ODK and ADL status

The ODK/pa/,/ta/, and/ka/were lower in the poor ADL group (all *p* < 0.05). The univariate logistic regression analyses showed that poorer ODK performance (/pa/,/ta/, and/ka/) was associated with poorer ADL (Table 2). In the age-adjusted models, lower ODK performance remained associated with poor ADL. A one-repetition-per-second decrease in ODK was associated with 1.40- to 1.45-fold higher odds of being in the poor ADL group. However, the association was modest, with an imprecise 95% confidence interval (1.06–1.07).

**Table 2:** Logistic regression analysis of factors associated with poor ADL (BI < 90)

Variable	Crude model		Age-adjusted model	
	OR (95% CI)	*p*-value	OR (95% CI)	*p*-value
Tongue pressure (per 1 kPa decrease)	1.03 (0.98–1.09)	0.26	1.03 (0.98–1.09)	0.27
ODK/pa/(per 1 reps/s decrease)	1.41 (1.07–2.01)	< 0.05	1.40 (1.00–2.18)	< 0.05
ODK/ta/(per 1 reps/s decrease)	1.45 (1.07–2.06)	< 0.05	1.43 (1.01–2.10)	< 0.05
ODK/ka/(per 1 reps/s decrease)	1.47 (1.06–2.12)	< 0.05	1.45 (1.03–2.17)	< 0.05
Masticatory performance (per 1 score decrease)	1.28 (1.02–1.75)	< 0.05	1.28 (0.97–1.89)	0.09
Present teeth (per 1 tooth decrease)	1.04 (0.99–1.10)	0.17	1.03 (0.98–1.10)	0.23
Functional teeth (per 1 tooth decrease)	1.12 (0.96–1.23)	< 0.05	1.12 (0.97–1.24)	< 0.05

95% CIs were estimated using bootstrap resampling with 1000 iterations (percentile method)

*OR* Odds ratio, *CI* Confidence interval, *ADL* Activities of daily living, *BI* Barthel Index, *ODK* Oral diadochokinesis

In the sensitivity analysis using BI as a continuous variable, all ODK measures (/pa/,/ta/, and/ka/) were associated with ADL after adjustment for age (Table S1, Additional file 1), consistent with the findings from the logistic regression analyses. The standardized regression coefficients (β) ranged from 0.25 to 0.28.

### Associations of tongue pressure, masticatory performance, and number of teeth with ADL status

Tongue pressure did not differ between the two groups. Masticatory performance was lower in the poor ADL group (*p* < 0.05). The univariate logistic regression analyses showed that lower masticatory performance was associated with poor ADL (Table 2); in particular, higher odds of poor ADL were observed per one-point decrease in the masticatory performance score. However, this association disappeared in the age-adjusted models. Neither the number of present teeth nor the number of functional teeth was associated with ADL status.

### Correlations of masticatory performance with ODK

Masticatory performance was not correlated with ODK/pa/,/ta/, or/ka/(/pa/: *ρ* = 0.20, *p* = 0.05;/ta/: *ρ* = 0.18, p = 0.07;/ka/: *ρ* = 0.11, *p* = 0.26, respectively).

### ROC analyses

ROC analyses were performed to examine the discriminative ability of oral functions in detecting poor ADL (Table 3). The area under the curve (AUC) values for ODK/pa/,/ta/,/ka/, and masticatory performance were all in the range of 0.66–0.67, indicating only modest discrimination between good and poor ADL.

**Table 3:** ROC analysis for detecting poor ADL (BI < 90)

Variable	Cutoff value	AUC	95% CI	Sensitivity (%)	Specificity (%)	Youden index
ODK/pa/(reps/s)	3.9	0.66	0.53–0.78	84.8	42.9	0.28
ODK/ta/(reps/s)	4.1	0.67	0.54–0.79	75.9	52.4	0.28
ODK/ka/(reps/s)	5.7	0.66	0.54–0.78	29.1	95.2	0.24
Masticatory performance (score)	3.5	0.67	0.53–0.80	59.5	71.4	0.31

*ROC* Receiver operating characteristic, *AUC* Area under the curve, *CI* Confidence interval, *ADL* Activities of daily living, *ODK* Oral diadochokinesis

## Discussion

In this study, we examined the association between tongue and masticatory functions and ADL status and explored whether these measures, including ODK as a simple method for assessing oral neuromotor function, may be potential early indicators of ADL decline in older adults. Both ODK and masticatory performance were associated with ADL, and the association between ODK and poorer ADL remained even after adjusting for age. However, the discriminatory ability of ODK was limited, suggesting that its usefulness as a standalone screening tool may be restricted.

Studies have reported that reduced ODK and masticatory performance are associated with sarcopenia [7, 8], which is linked to physical impairment and poor ADL status [11, 12]. Consistent with these findings, the poor ADL group in the current study demonstrated a higher prevalence of handgrip weakness and reduced ODK and mastication performance.

In addition to this association with sarcopenia, few studies have directly investigated the relationship between tongue and masticatory functions and ADL. Kimura, et al. [13] reported that poor masticatory performance in community-dwelling older adults was associated with depressive symptoms, cognitive decline, and reduced intellectual activity. Studies in older adults requiring care or in patients with sarcopenic dysphagia have shown that ODK and tongue pressure are related to ADL decline [14, 23]. In the present study, which included participants with varying levels of ADL, tongue pressure was not related to ADL, but ODK was associated with ADL. To discriminate relatively independent individuals from those with at least mild functional limitations, a BI cutoff value of 90 was applied based on previous studies in Japanese older adults and to account for the ceiling effect observed in this relatively high-functioning population. The observed associations were modest, with the relatively wide confidence intervals indicating limited precision. This suggests that the association between ODK and ADL should be interpreted with caution. Given the small number of participants with poor ADL and the limited adjustment for potential confounders, the observed associations between ODK and ADL should be interpreted with caution and confirmed in larger, adequately powered studies.

ODK/pa/reflects lip movement and is reportedly correlated with gait speed among community-dwelling older adults [24]. Moreover, reduced ODK/pa/has been identified as a risk factor for dementia [25]. In contrast, ODK/ta/and/ka/reflect tongue movement. The production of/ta/requires anterior tongue elevation against the hard palate, whereas the production of/ka/requires posterior tongue elevation against the soft palate; therefore, both are closely related to tongue strength and swallowing function [26, 27]. Indeed,/ka/is reportedly associated with oral health-related quality of life, including eating and communication [28], balance ability [29], and cognitive function [30]. Similarly,/ta/is associated with gait speed [31] and physical pre-frailty in older adults [32]. Moreover, reduced ODK is linked to cognitive decline [30] and decreased social participation [33], both of which are associated with poor ADL [34, 35]. Collectively, these findings suggest that ODK is related to motor function, cognitive function, and ADL, which may reflect shared underlying functional processes.

In the ROC analyses, AUC values of 0.66–0.67 indicated poor discriminatory ability, suggesting that neither ODK nor masticatory performance is adequate as a stand-alone screening tool for detecting early ADL decline. Therefore, their clinical applicability as screening tools should be interpreted with caution. Nevertheless, their associations with ADL, even among older adults with only mild functional decline, suggest that oral function reflects aspects of functional status rather than serving as a screening tool. Longitudinal studies are needed to clarify whether ODK can potentially contribute to early identification and monitoring of ADL decline.

Consistent with this potential early indicator perspective, we found that participants with even mild ADL decline demonstrated median values below the established thresholds for oral hypofunction (ODK < 6 repetitions/s; masticatory performance score < 2). However, given the limited discriminatory ability observed, these findings should be interpreted cautiously. Oral function is essential not only for adequate nutrition but also for speech and effective communication, and its decline has been associated with reduced social participation [33, 36, 37] and lower quality of life [38, 39]. Previous studies suggested that oral and masticatory functions can be improved through interventions such as oral exercises and comprehensive programs that address both oral function and nutrition [40–42]. However, the clinical implications of the present findings remain exploratory.

This study has a few limitations. First, although dichotomizing BI at 90 was data-driven (ceiling effect), it may have resulted in loss of information and potential misclassification. Future studies should consider using continuous BI scores or lower cut-off values to better capture severe disability. Second, the small number of participants in the poor-ADL group limited the ability to adjust for potential confounders. Therefore, the observed association between ODK and ADL may partly reflect underlying systemic factors, such as overall physical or nutritional status, rather than an independent effect of oral function. Although we adjusted only for age in the multivariable models, future studies with larger samples should include these variables to account for confounding factors. Third, the cross-sectional design precludes any causal inferences. Fourth, because relatively few participants had BI scores below 90, we were unable to fully examine whether the observed associations applied to older adults with more pronounced ADL impairment (e.g., BI ≤ 85). Future studies should include a larger number of participants with lower BI scores to clarify the generalizability of our findings across the full spectrum of ADL disability. Finally, the inclusion of participants from both community settings and supported residential environments may have introduced heterogeneity in ADL and oral function, as differences in support environments may have influenced the observed associations.

## Conclusions

ODK/pa/,/ta/, and/ka/were associated with ADL status, even among older adults who showed no or only mild decline in ADL performance. However, the strength of these associations was modest, showing that ODK has limited discriminatory ability as a screening tool for ADL decline. These findings suggest that oral motor function may be a potential early indicator of functional vulnerability. Future longitudinal studies are warranted to validate these findings.

## Supplementary Information

Additional file 1: Table S1. Linear regression analyses of the association between ODK and the Barthel Index.

## Data Availability

The data that support the findings of this study are available on request from the corresponding author. The data are not publicly available due to privacy or ethical restrictions.

## Abbreviations

ADL: Activities of daily living
AUC: Area under the curve
BI: Barthel Index
BMI: Body mass index
ODK: Oral diadochokinesis
CI: Confidence interval
OR: Odds ratio
ROC: Receiver operating characteristic
IQR: Interquartile range
MNA-SF: Mini Nutritional Assessment–Short Form
