# Supplementary material for: Association of oral diadochokinesis with activities of daily living in older adults: a cross-sectional observational study
Source: BMC Oral Health. 2026 Jun 3;26:1567. doi: 10.1186/s12903-026-08799-1 (PMC13505105; doi:10.1186/s12903-026-08799-1)
Supplement: Supplementary file 1 [file 12903_2026_8799_MOESM1_ESM.docx]

Table S1. Linear regression analyses of the association between ODK and the Barthel Index

|  | Model 1 | | |  | Model 2 | | | |
| --- | --- | --- | --- | --- | --- | --- | --- | --- |
| Variable (reps/s) | β | B (95% CI) | p-value |  | β | B (95% CI) | p-value | Adjusted R² |
| ODK /pa/ | 0.25 | 2.16(0.05–3.81) | < 0.05 |  | 0.25 | 2.18(0.48–3.87) | < 0.05 | 0.04 |
| ODK /ta/ | 0.26 | 2.26(0.59–3.93) | < 0.01 |  | 0.27 | 2.29(0.58–4.00) | < 0.01 | 0.05 |
| ODK /ka/ | 0.28 | 2.38(0.72–4.05) | < 0.01 |  | 0.28 | 2.39(0.70–4.09) | < 0.01 | 0.06 |

The Barthel Index was analyzed as a continuous variable.
β = standardized regression coefficient; B = unstandardized regression coefficient; CI = confidence interval; ODK = oral diadochokinesis.
Model 1: Crude model.
Model 2: Adjusted for age.
The overall models showed statistical evidence of association (p < 0.05).
